# Supplementary material for: Genetic Analysis of Early White Quality Protein Maize Inbreds and Derived Hybrids under Low-Nitrogen and Combined Drought and Heat Stress Environments
Source: Plants (Basel). 2021 Nov 26;10(12):2596. doi: 10.3390/plants10122596 (PMC8706249; doi:10.3390/plants10122596)
Supplement: Supplementary file 1 [file plants-10-02596-s001.zip › plants-1453433-supplementary.pdf]

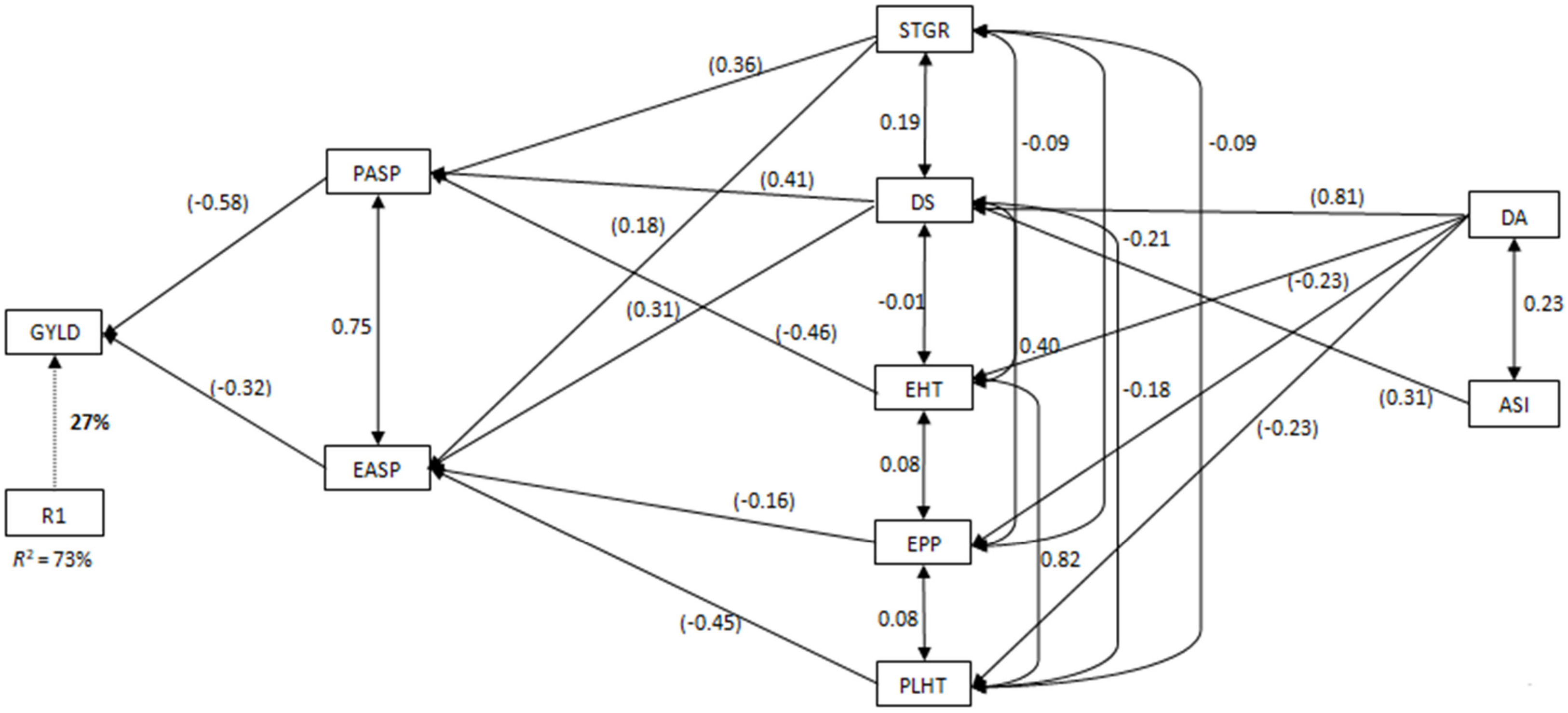

Supplementary Figure S1: Path analysis model diagram showing causal relationships of measured traits of early maturing QPM hybrids evaluated under low-N in 2019 and 2020 growing seasons at Mokwa in Nigeria. Bold value is the residual effect; values in parenthesis are direct path coeeficients while other values are correlation coefficients. R^2 = coefficient of determination; R1= residual effects; GYLD = Grain yield; DA= days to 50% anthesis; DS = days to 50% silking; ASI = antheisi-silking interval; PLHT = plant height; EHT = ear height; PASP = plant aspect; EASP = ear aspect; EPP = ears per plot; STGR = stay green characteristic

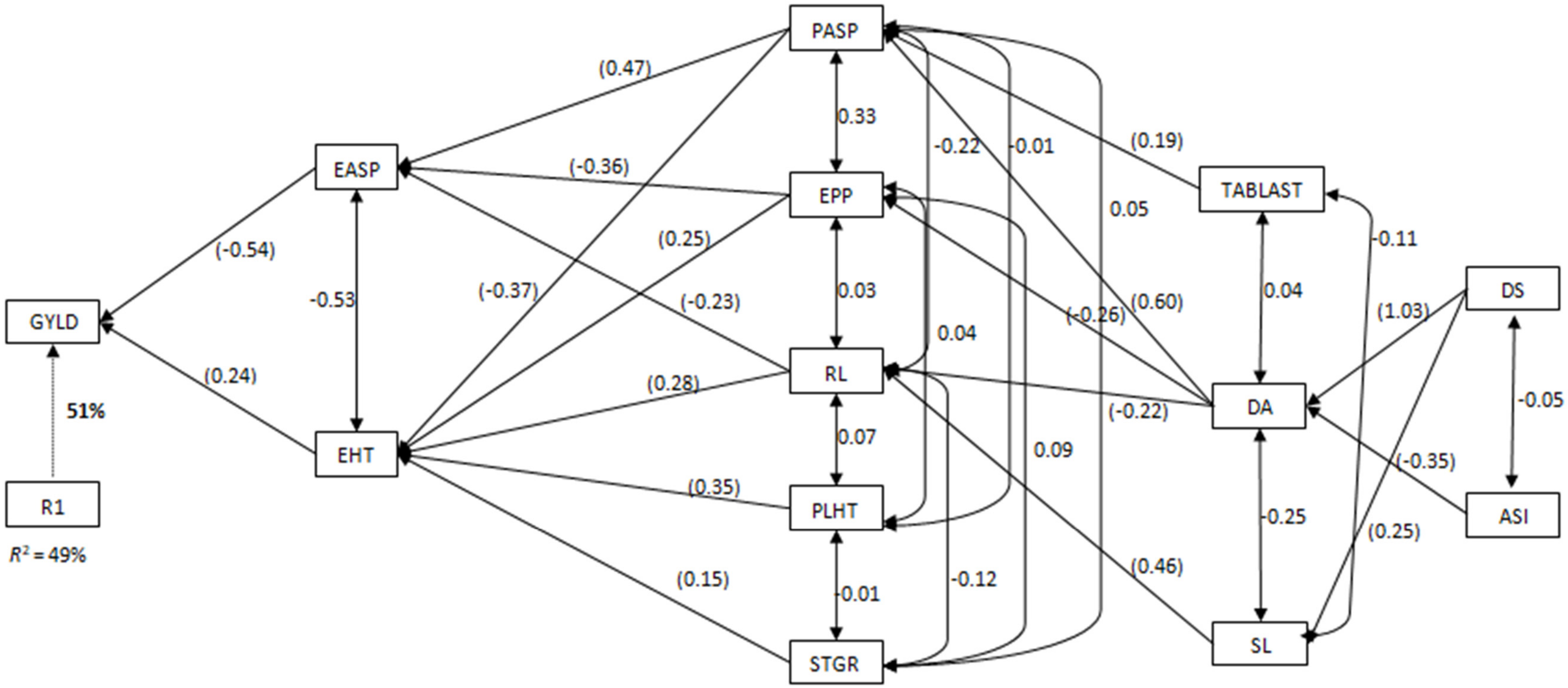

Supplementary Figure S2: Path analysis model diagram showing causal relationships of measured traits of early maturing QPM hybrids evaluated under CDHS in 2020 and 2021 growing seasons at Kadawa in Nigeria. Bold value is the residual effect; values in parenthesis are direct path coeeficients while other values are correlation coefficients. R^2 = coefficient of determination; R1= residual effects; GYLD = Grain yield; DA= days to 50% anthesis; DS = days to 50% silking; ASI = antheisi-silking interval; PLHT = plant height; EHT = ear height; PASP = plant aspect; EASP = ear aspect; EPP = ears per plot; STGR = stay green characteristic; RL = root lodging; SL = stalk lodging; TABLAST = tassel blast

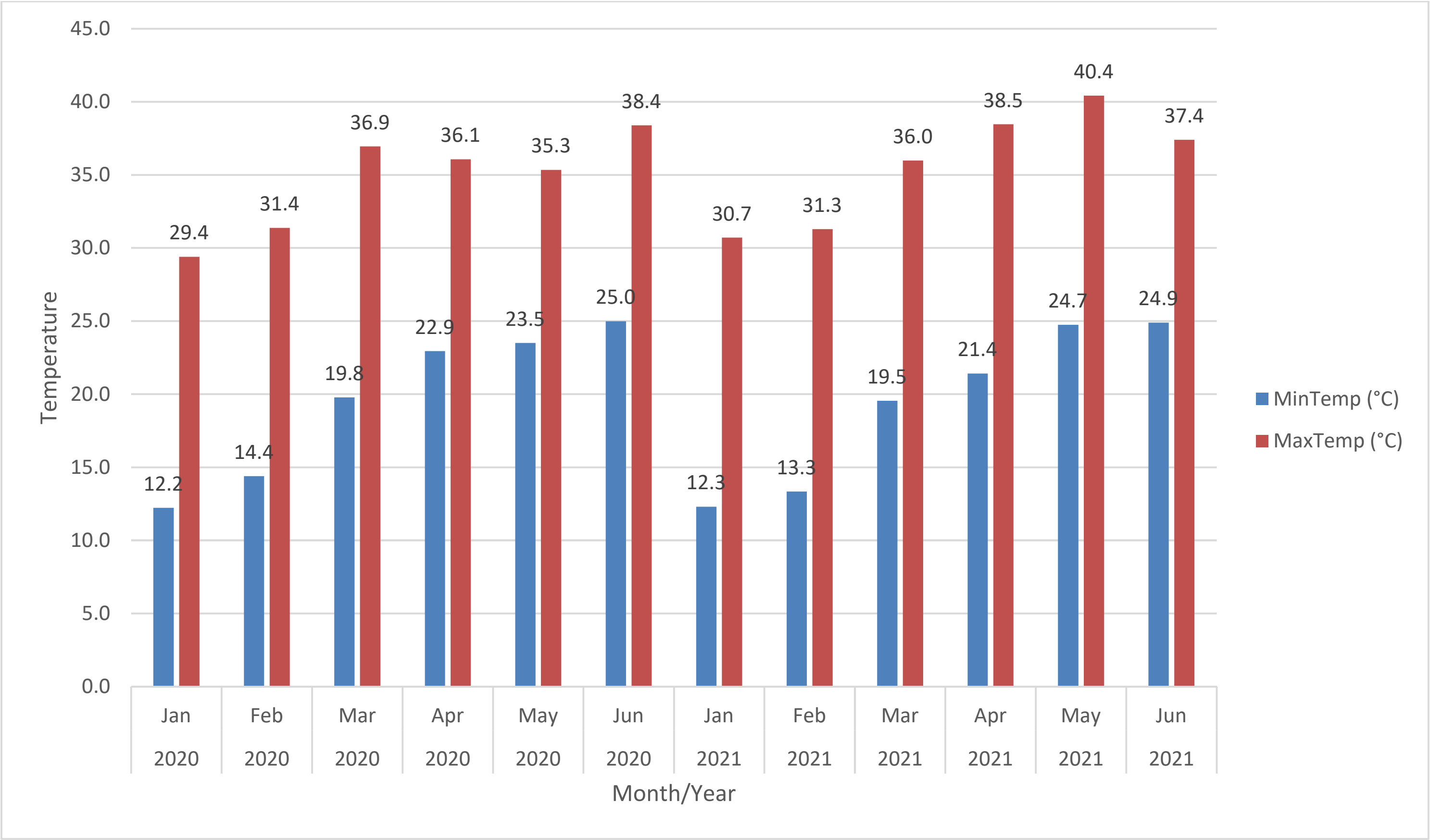

Supplementary Figure S3: Monthly average temperature during the experimental period in 2020 and 2021 at Kadawa, Nigeria

Supplementary Table S1: North Carolina Design II mating design among 24 early maturing quality protein maize inbred lines.

	Males			
**Females**		Set F		
Set A	TZEQI 240	TZEQI 241	TZEQI 246	TZEQI 6
TZEQI 106	106×240	106×241	106×246	106×6
TZEQI 113	113×240	113×241	113×246	113×6
TZEQI 122	122×240	122×241	122×246	122×6
TZEQI 123	123×240	123×241	123×246	123×6
		Set A		
Set B	TZEQI 106	TZEQI 113	TZEQI 122	TZEQI 123
TZEQI 130	130×106	130×113	130×122	130×123
TZEQI 132	132×106	132×113	132×122	132×123
TZEQI 140	140×106	140×113	140×122	140×123
TZEQI 143	143×106	143×113	143×122	143×123
		Set B		
Set C	TZEQI 130	TZEQI 132	TZEQI 140	TZEQI 143
TZEQI 158	158×130	158×132	158×140	158×143
TZEQI 159	159×130	159×132	159×140	159×143
TZEQI 162	162×130	162×132	162×140	162×143
TZEQI 165	165×130	165×132	165×140	165×143
		Set C		
Set D	TZEQI 158	TZEQI 159	TZEQI 162	TZEQI 165
TZEQI 171	171×158	171×158	171×162	171×165
TZEQI 175	175×158	175×158	175×162	175×165
TZEQI 176	176×158	176×158	176×162	176×165
TZEQI 188	188×158	188×158	188×162	188×165
		Set D		
Set E	TZEQI 171	TZEQI 175	TZEQI 176	TZEQI 188
TZEQI 210	210×171	210×175	210×176	210×188
TZEQI 216	216×171	216×175	216×176	216×188
TZEQI 219	219×171	219×175	219×176	219×188
TZEQI 228	228×171	228×175	228×176	228×188
		Set E		
Set F	TZEQI 210	TZEQI 216	TZEQI 219	TZEQI 228
TZEQI 240	240×210	240×216	240×219	240×228
TZEQI 241	241×210	241×216	241×219	241×228
TZEQI 246	246×210	246×216	246×219	246×228
TZEQI 6	6×210	6×216	6×219	6×228

Supplementary Table S2: Daily minimum and maximum tempearture at Kadawa 2020 dry season.

Day	Month	Year	MinTemp	Max Temp
			(° C)	(° C
1	Feb	2020	12.5	29.1
2	Feb	2020	13.4	30.2
3	Feb	2020	11.1	30.1
4	Feb	2020	13.0	29.2
5	Feb	2020	12.7	29.3
6	Feb	2020	12.1	29.8
7	Feb	2020	12.9	31.8
8	Feb	2020	13.7	31.4
9	Feb	2020	16.1	32.0
10	Feb	2020	17.5	31.9
11	Feb	2020	14.8	29.5
12	Feb	2020	12.5	28.0
13	Feb	2020	13.4	27.9
14	Feb	2020	12.3	31.7
15	Feb	2020	11.5	32.3
16	Feb	2020	12.1	33.3
17	Feb	2020	14.5	31.4
18	Feb	2020	15.1	30.7
19	Feb	2020	15.2	31.4
20	Feb	2020	14.9	31.3
21	Feb	2020	16.2	33.4
22	Feb	2020	16.5	34.3
23	Feb	2020	16.0	33.0
24	Feb	2020	15.5	31.0
25	Feb	2020	14.9	30.9
26	Feb	2020	14.2	32.0
27	Feb	2020	16.0	34.4
28	Feb	2020	16.9	33.7
29	Feb	2020	20.1	35.0
1	March	2020	19.1	37.1
2	March	2020	19.5	36.0
3	March	2020	20.0	36.0
4	March	2020	20.4	37.4
5	March	2020	18.4	37.6
6	March	2020	16.9	37.0
7	March	2020	18.0	37.3
8	March	2020	21.8	37.6
9	March	2020	19.1	38.3
10	March	2020	18.4	38.8
11	March	2020	22.5	37.6
12	March	2020	19.6	30.8
13	March	2020	15.2	32.6
14	March	2020	15.1	33.2
15	March	2020	14.9	34.7
16	March	2020	15.3	36.4
17	March	2020	16.0	37.8
18	March	2020	17.8	38.2
19	March	2020	19.2	38.9
20	March	2020	20.3	38.8

21	March	2020	20.6	39.8
22	March	2020	21.4	40.5
23	March	2020	22.9	40.1
24	March	2020	24.0	31.3
25	March	2020	22.9	32.9
26	March	2020	22.7	36.6
27	March	2020	21.9	37.8
28	March	2020	22.9	39.1
29	March	2020	21.3	38.5
30	March	2020	22.5	39.0
31	March	2020	22.8	37.7
1	April	2020	23.3	35.3
2	April	2020	24.2	34.8
3	April	2020	24.6	37.0
4	April	2020	24.1	38.1
5	April	2020	24.7	36.2
6	April	2020	24.5	37.0
7	April	2020	24.3	29.8
8	April	2020	22.8	32.9
9	April	2020	23.2	31.1
10	April	2020	23.8	32.0
11	April	2020	21.3	34.6
12	April	2020	21.0	33.0
13	April	2020	21.0	37.0
14	April	2020	20.3	37.5
15	April	2020	19.9	37.9
16	April	2020	20.5	38.4
17	April	2020	20.3	38.4
18	April	2020	18.9	38.5
19	April	2020	21.3	38.4
20	April	2020	22.4	37.4
21	April	2020	23.7	38.0
22	April	2020	23.7	38.4
23	April	2020	23.6	39.0
24	April	2020	23.7	38.6
25	April	2020	24.3	38.0
26	April	2020	24.7	36.2
27	April	2020	24.6	34.2
28	April	2020	24.4	36.6
29	April	2020	25.0	35.4
30	April	2020	24.5	32.8
1	May	2020	23.7	36.2
2	May	2020	24.2	37.4
3	May	2020	25.2	33.2
4	May	2020	23.9	34.3
5	May	2020	23.0	38.4
6	May	2020	22.4	38.3
7	May	2020	22.3	38.1
8	May	2020	24.0	38.9
9	May	2020	24.4	35.0
10	May	2020	23.0	34.2
11	May	2020	22.7	33.1
12	May	2020	22.9	35.7

13	May	2020	23.1	37.6
14	May	2020	22.0	38.4
15	May	2020	23.8	35.4
16	May	2020	24.0	34.6
17	May	2020	24.1	34.0
18	May	2020	23.5	37.0
19	May	2020	25.4	36.1
20	May	2020	24.2	33.6
21	May	2020	22.2	35.6
22	May	2020	23.0	31.3
23	May	2020	22.9	33.6
24	May	2020	22.6	31.3
25	May	2020	23.4	33.1
26	May	2020	23.8	33.7
27	May	2020	23.5	32.3
28	May	2020	23.7	35.2
29	May	2020	23.0	36.6
30	May	2020	24.1	33.2
31	May	2020	24.6	40.5
1	June	2020	25.9	41.1
2	June	2020	25.7	40.9
3	June	2020	24.3	40.7
4	June	2020	25.8	38.5
5	June	2020	25.0	40.7
6	June	2020	25.2	40.9
7	June	2020	25.7	40.0
8	June	2020	24.6	41.1
9	June	2020	25.9	38.2
10	June	2020	24.6	37.8
11	June	2020	24.8	38.2
12	June	2020	26.0	39.6
13	June	2020	25.8	39.8
14	June	2020	26.0	39.0
15	June	2020	24.4	40.6
16	June	2020	26.6	39.0
17	June	2020	25.8	34.8
18	June	2020	24.7	34.3
19	June	2020	24.2	35.4
20	June	2020	23.9	38.0
21	June	2020	24.1	39.1
22	June	2020	24.2	36.1
23	June	2020	24.6	35.7
24	June	2020	24.1	39.5
25	June	2020	25.0	40.1
26	June	2020	24.2	37.9
27	June	2020	25.1	36.2
28	June	2020	24.5	37.4
29	June	2020	24.5	36.1
30	June	2020	24.7	35.1

Supplementary Table S3: Daily minimum and maximum tempearture at Kadawa 2021 dry season.

Day	Month	Year	MinTemp (° C)	Max Temp (° C
1	Feb	2021	18.4	33.7
2	Feb	2021	21.1	35.1
3	Feb	2021	19.9	35.2
4	Feb	2021	16.7	34.5
5	Feb	2021	17.4	34.4
6	Feb	2021	17.2	34.5
7	Feb	2021	16.8	34.6
8	Feb	2021	18.2	33.1
9	Feb	2021	17.2	34.7
10	Feb	2021	16.5	35.2
11	Feb	2021	16.2	35.2
12	Feb	2021	16.5	35.7
13	Feb	2021	17.3	35.7
14	Feb	2021	19.8	36.2
15	Feb	2021	20.0	36.4
16	Feb	2021	17.4	36.1
17	Feb	2021	17.1	33.4
18	Feb	2021	17.0	31.3
19	Feb	2021	14.6	31.3
20	Feb	2021	16.1	33.4
21	Feb	2021	17.8	34.1
22	Feb	2021	18.5	34.6
23	Feb	2021	17.5	35.1
24	Feb	2021	16.9	35.4
25	Feb	2021	18.9	35.4
26	Feb	2021	22.3	35.4
27	Feb	2021	18.6	36.3
28	Feb	2021	19.4	37.2
1	Mar	2021	19.3	36.8
2	Mar	2021	19.0	37.2
3	Mar	2021	19.8	37.6
4	Mar	2021	18.8	37.8
5	Mar	2021	19.1	38.1
6	Mar	2021	19.8	38.5
7	Mar	2021	20.4	38.1
8	Mar	2021	20.8	38.3
9	Mar	2021	22.2	39.1
10	Mar	2021	19.5	37.8
11	Mar	2021	23.0	30.9
12	Mar	2021	22.6	29.9
13	Mar	2021	23.0	35.8
14	Mar	2021	23.1	31.5
15	Mar	2021	21.7	28.8
16	Mar	2021	21.8	32.4
17	Mar	2021	22.0	33.2
18	Mar	2021	21.7	35.7
19	Mar	2021	23.1	31.5
20	Mar	2021	22.0	30.2
21	Mar	2021	22.9	34.7

22	Mar	2021	23.9	35.8
23	Mar	2021	22.4	36.6
24	Mar	2021	22.3	28.4
25	Mar	2021	21.7	30.6
26	Mar	2021	21.1	34.4
27	Mar	2021	20.4	35.3
28	Mar	2021	18.6	34.8
29	Mar	2021	20.0	34.5
30	Mar	2021	18.3	34.5
31	Mar	2021	17.8	35.6
1	Apr	2021	18.8	36.3
2	Apr	2021	19.3	36.8
3	Apr	2021	19.4	37.0
4	Apr	2021	22.6	35.7
5	Apr	2021	21.7	38.9
6	Apr	2021	22.7	39.5
7	Apr	2021	23.2	37.4
8	Apr	2021	22.6	39.3
9	Apr	2021	23.2	35.2
10	Apr	2021	23.0	31.8
11	Apr	2021	23.5	35.6
12	Apr	2021	22.9	33.0
13	Apr	2021	22.8	30.9
14	Apr	2021	21.7	33.5
15	Apr	2021	21.2	34.4
16	Apr	2021	22.3	35.0
17	Apr	2021	22.4	30.1
18	Apr	2021	22.6	32.8
19	Apr	2021	21.8	36.4
20	Apr	2021	22.6	34.7
21	Apr	2021	23.2	35.5
22	Apr	2021	23.4	31.3
23	Apr	2021	22.9	31.5
24	Apr	2021	23.1	32.9
25	Apr	2021	22.3	30.6
26	Apr	2021	21.2	35.3
27	Apr	2021	23.3	30.0
28	Apr	2021	22.6	33.8
29	Apr	2021	22.5	35.1
30	Apr	2021	23.1	32.4
1	May	2021	22.2	29.5
2	May	2021	21.5	35.7
3	May	2021	22.8	30.1
4	May	2021	21.8	30.6
5	May	2021	21.2	31.7
6	May	2021	21.3	32.4
7	May	2021	21.3	27.3
8	May	2021	20.7	29.1
9	May	2021	21.1	28.8
10	May	2021	20.2	33.6
11	May	2021	23.5	34.2
12	May	2021	22.8	30.0
13	May	2021	22.2	27.5

14	May	2021	20.9	30.5
15	May	2021	21.7	31.4
16	May	2021	21.8	31.3
17	May	2021	22.2	27.4
18	May	2021	21.1	31.0
19	May	2021	20.9	33.3
20	May	2021	21.2	31.9
21	May	2021	22.4	29.4
22	May	2021	22.1	30.4
23	May	2021	22.1	33.1
24	May	2021	22.5	31.0
25	May	2021	20.6	33.8
26	May	2021	22.0	28.0
27	May	2021	21.5	31.2
28	May	2021	21.1	33.1
29	May	2021	22.1	33.4
30	May	2021	22.3	35.3
31	May	2021	22.9	27.5
1	June	2021	22.3	29.1
2	June	2021	21.6	29.9
3	June	2021	21.1	31.4
4	June	2021	22.2	28.5
5	June	2021	20.5	29.5
6	June	2021	20.7	29.4
7	June	2021	19.3	30.2
8	June	2021	21.4	32.1
9	June	2021	21.9	32.0
10	June	2021	20.7	33.0
11	June	2021	22.4	31.6
12	June	2021	22.0	27.7
13	June	2021	21.5	26.3
14	June	2021	20.5	30.4
15	June	2021	20.8	29.0
16	June	2021	21.5	28.3
17	June	2021	21.3	26.9
18	June	2021	20.2	28.0
19	June	2021	20.3	29.6
20	June	2021	21.3	27.3
21	June	2021	20.6	28.6
22	June	2021	21.3	26.8
23	June	2021	20.4	24.5
24	June	2021	19.8	27.5
25	June	2021	19.6	28.4
26	June	2021	21.5	28.2
27	June	2021	21.2	25.8
28	June	2021	20.9	25.1
29	June	2021	20.3	26.5
30	June	2021	19.9	26.8
